# Whither thiotepa (for patients in the USA)?

**DOI:** 10.1007/s11060-015-1856-4

**Published:** 2015-07-26

**Authors:** Christopher Dardis, Kelly Milton, Lynn Ashby

**Affiliations:** Department of Neurology, Barrow Neurological Institute, Suite 300, 500 W Thomas Road, Phoenix, AS 85014 USA

To the Editor,

We wish to highlight the ongoing shortage of thiotepa in the USA so that our colleagues may plan ahead accordingly. We are not aware of similar shortages of this agent affecting other countries.

In neuro-oncology, thiotepa is most often used for the treatment of leptomeningeal metastases. It is recommended for this indication in the most recently issued National Comprehensive Cancer Network (NCCN) Guidelines.

In the Guidelines, it appears fourth in a list of nine, just below (liposomal) cytarabine and methotrexate [1]. Along with topotecan and etoposide, it is one of *only* *three* agents which is indicated by general use (e.g. as opposed to methotrexate where its use is specifically recommended for lymphoma/leukemia and for breast cancer). Like most of the agents on the list, its patent has expired.

Thiotepa has been approved by the US Food and Drug Administration (FDA) for:

- bladder cancer (intravesical)
- ovarian and breast cancer (intravenous)
- malignant effusions (locally).

In all three conditions, it has been superseded by other treatments and may be considered at best a ‘third-line’ agent. To our knowledge, approval has not been sought for intrathecal use.

In the USA, the supply shortage came about in July 2014 following the acquisition of the product from the former manufacturer, Bedford Laboratories, by Hikma Pharmaceuticals. Currently, the only available FDA-approved distributor, Adienne SA, exports from Switzerland. The manufacture occurs in Australia.

Pharmacies in the USA (including those which are hospital-based) are unlikely to stock this medicine routinely and will need some advance notice if the drug is required. Based on our experience, we expect a delay of 3–4 weeks between ordering and receiving thiotepa. We became aware of the shortage while treating a patient with ovarian cancer with leptomeningeal metastases. We favored thiotepa as it is has been used for systemic treatment of this condition.

Where use is expected in the upcoming year, it is important to have reserves available. We suggest keeping 8–10 × 15 mg vials on hand. These can be reconstituted locally to make the standard dose of 10 mg, which is given twice/week. This should allow sufficient time for additional supplies to arrive, if required. Storage of thiotepa is only recommended for 1 year, so additional supplies may need to be ordered on an annual basis.

Drug availability is a particular concern in this field, where many of the agents commonly used no longer have an active patent. Other examples are memantine and dexamethasone (IV), both of which are listed on the FDA’s Drug Shortages Database as “Currently in Shortage” at the time of writing [2]. To help the practitioner keep track of such shortages, the FDA has recently developed a mobile application [3].

## References

[1] National Comprehensive Cancer Network and others (2014) NCCN clinical practice guidelines in oncology: central nervous system cancers. Version 2 10.6004/jnccn.2022.0062 36509072

[2] U.S. Food and Drug Administration (2015) FDA drug shortages. http://www.accessdata.fda.gov/scripts/drugshortages/. Accessed 4 Apr 2015

[3] U.S. Food and Drug Administration (2015) FDA launches drug shortages mobile app. http://www.fda.gov/NewsEvents/Newsroom/PressAnnouncements/ucm436481.htm. Accessed 10 Apr 2015

